# Variability in the reported management of pulmonary metastases in osteosarcoma

**DOI:** 10.1002/cam4.407

**Published:** 2015-01-28

**Authors:** Onita Bhattasali, Andrea T Vo, Michael Roth, David Geller, R Lor Randall, Richard Gorlick, Jonathan Gill

**Affiliations:** 1 Division of Pediatric Hematology/Oncology, Children's Hospital at Montefiore, Albert Einstein College of MedicineBronx, New York; 2 Department of Orthopaedic Surgery, Montefiore Medical Center and the Children's Hospital at Montefiore, Albert Einstein College of MedicineBronx, New York; 3 Sarcoma Services, Huntsman Cancer Institute, University of UtahSalt Lake City, Utah; 4 Department of Molecular Pharmacology, Albert Einstein College of MedicineBronx, New York

**Keywords:** Osteosarcoma, pulmonary metastases, practice patterns

## Abstract

Nearly 20% of patients with newly diagnosed osteosarcoma have detectable metastases at diagnosis; the majority of which occur in the lungs. There are no established recommendations for the timing and modality of metastasectomy. Members of the Connective Tissue Oncology Society (CTOS) were emailed an anonymous 10-min survey assessing their management practices for pulmonary findings at the time of an osteosarcoma diagnosis. The questionnaire presented three scenarios and discussed the choice to perform surgery, the timing of resection, and the choice of surgical procedure. Analyses were stratified by medical profession. One hundred and eighty-three physicians responded to our questionnaire. Respondents were comprised of orthopedic surgeons (37%), medical oncologists (31%), pediatric oncologists (22%), and other medical subspecialties (10%). There was variability among the respondents in the management of the pulmonary nodules. The majority of physicians chose to resect the pulmonary nodules following neoadjuvant chemotherapy (46–63%). Thoracotomy was the preferred technique for surgical resection. When only unilateral findings were present, the majority of physicians did not explore the contralateral lung. The majority of respondents did not recommend resection if the pulmonary nodule disappeared following chemotherapy. The survey demonstrated heterogeneity in the management of pulmonary metastases in osteosarcoma. Prospective trials need to evaluate whether these differences in management have implications for outcomes for patients with metastatic osteosarcoma.

## Introduction

Osteosarcoma is the most common primary malignant tumor of bone in children and young adults. At the time of diagnosis 10–15% of patients have detectable metastases, of which ∽90% occur in the lungs 1,2. The presence of metastatic disease at presentation portends a worse survival of 30% as compared to greater than 70% for patients with localized disease 2,3. For patients with localized disease standard treatment involves extirpation of the primary tumor in conjunction with chemotherapy. The long-term survival of patients with retained macroscopic tumor, involving the primary site or metastases, is less than 10% 1.

Aggressive resection of all pulmonary disease has been shown to prolong survival and be necessary for curative intent. A retrospective, single-institution review of patients treated at MD Anderson Cancer Center demonstrated that patients who underwent resection of pulmonary metastases had a mean survival of 33 months compared to 10 months for those who did not undergo resection 4. However, the major indication for not undergoing resection was the determination of having unresectable disease. The differences in survival may represent underlying disparities in the cohorts as those that did not undergo resection may have had more advanced disease or more aggressive biology.

Another confounding variable is the lack of clarity of what represents metastatic disease. In the MD Anderson review greater than 5% of the patients who underwent resection were found not to have osteosarcoma metastases 4. The most frequently used modality for defining metastatic disease is the visualization of nodules on computed tomography (CT) of the chest. Due to the limits of radiographic imaging, some osteosarcoma lesions are palpable even though they have not been previously visualized. Kayton, et al. found in a retrospective comparison between CT imaging and pathologic review that CT underestimated the number of metastases in 35%, overestimated in 37%, and demonstrated concordance in 28% of the cases. Of the 329 lung nodules resected in 54 thoracotomies only 63.5% were pathologically confirmed to be osteosarcoma metastases. Fifteen percent of the thoracotomies did not demonstrate any pathologically confirmed osteosarcoma despite the imaging findings 5. In contrast, patients who present with unilateral metastases on CT imaging frequently have palpable disease on exploration of the contralateral lung 6.

It is important to note, however, that imaging technology has considerably improved in the time period following the publication of these studies, and radiologists are now better equipped to identify pulmonary metastases in osteosarcoma. More recently, a review by Brader, et al. of 117 nodules demonstrated greater than 93% accuracy in identification of malignant nodules using independent predictors of malignant risk including calcifications and size greater than 5 mm 7. However, radiologists from the study were unable to accurately identify benign nodules and mislabeled over 70% of benign nodules as malignant suggesting that a large portion of patients may continue to be at risk of morbidity from unnecessary thoracic procedures.

There is also a lack of consensus regarding the clinical relevance of palpable osteosarcoma metastases that are not detectable on CT imaging. Karplus, et al., in a retrospective review of patients at St. Jude who underwent unilateral thoracotomy for unilateral disease, found that early relapses, occurring within 6 months, recurred with equal likelihood in the ipsilateral (11%) versus the contralateral (12%) lung. Overall, following unilateral resection, 17% of patients recurred on the ipsilateral versus 27% on the contralateral side, and 3% developed bilateral metastases 8. There was no statistical difference in these groups, leading the authors to conclude that occult metastases may have uncertain clinical significance.

Given the lack of definitive evidence to define practice patterns for patients with metastatic disease the recommendation from the National Comprehensive Cancer Network (NCCN) guidelines, clinical trials, and expert opinion is, when feasible, to completely resect all metastatic disease. There are no consensus recommendations for what defines metastatic disease in osteosarcoma, the timing of resection, or the surgical approach. To define normative practice patterns, the authors report the results of an online questionnaire addressed to members of the Connective Tissue Oncology Society (CTOS) regarding their views on the surgical management of pulmonary metastases in osteosarcoma.

## Materials and Methods

### Study population

The Connective Tissue Oncology Society (CTOS) is an organization dedicated to improving outcomes for patients with tumors of the connective tissue. The society consists of pediatric oncologists, medical oncologists, surgical oncologists, radiation oncologists, orthopedic surgeons, cardiothoracic surgeons, pathologists, radiologists, and scientists and has over 400 members. Members of CTOS were emailed a link to an anonymous survey regarding their views on the surgical management of pulmonary metastases in osteosarcoma. Only one response could be submitted from a given IP address. This study was approved by the Institutional Review Board of the Montefiore Medical Center and the Albert Einstein College of Medicine.

### Questionnaire

Participants were asked questions regarding their management decisions for pulmonary metastases in osteosarcoma. The questionnaire used a “choose your own adventure” format with follow-up questions varying based on the responses to prior questions. The questionnaire focused on three scenarios in which an 18-year-old boy presents with a new diagnosis of osteosarcoma of the left distal femur and pulmonary nodules on imaging: a single unilateral 2 cm nodule, a single unilateral 0.5 cm nodule, and multiple bilateral nodules.

The primary outcome was the decisions related to the management of the pulmonary nodules in each scenario. Respondents were interrogated regarding their views in each scenario for the need for resection, whether response to chemotherapy influenced their views, the timing of resection, surgical approach including thoracoscopy, thoracotomy, sternotomy, and the need for exploration of the contralateral lung. Secondary outcomes included variability in management as they pertained to demographic characteristics.

### Statistical methods

Descriptive statistics were computed for participant demographic characteristics. Bivariate analyses involving management decisions and physician characteristics were evaluated by *t-*test for continuous variables and chi-squared test for categorical variables.

## Results

### Demographic data

One hundred and eighty-three participants completed the questionnaire. Respondents included orthopedic surgeons (37%), medical oncologists (31%), pediatric oncologists (22%), radiation oncologists (5%), pathologists (2%), and pediatric surgeons, surgical oncologists, and “other” (each totaling 1%). Fifty-four percent of physicians reported completion of medical training in the United States, and 40% completed training outside of the United States. The majority of respondents practiced in an academic medical center (60%) or a cancer center (26%). Physicians were practicing for a median of 10–20 years and saw a median of 8–10 osteosarcoma patients a year with wide distribution in both categories. Most commonly the respondents (43%) reported that national guidelines were the predominant factors guiding their clinical management decisions (Table1).

**Table 1:** Demographics

Medical discipline		
Pediatric oncologist	41	22%
Medical oncologist	57	31%
Orthopedic surgeon	68	37%
Pediatric surgeon	1	1%
Radiation oncologist	9	5%
Surgical oncologist	2	1%
Pathologist	3	2%
Other/skipped	2	1%
Years from training		
0–5	28	15%
5–10	42	23%
10–20	51	28%
20+	51	28%
Skipped	8	4%
Medical education		
United States	98	54%
Outside US	74	40%
Skipped	11	6%
Location of current practice		
Cancer Center	48	26%
Academic medical center	110	60%
Private practice affiliated with academic medical center	2	1%
Private practice unaffiliated with academic medical center	8	4%
Other	1	1%
Skipped	14	8%
Number of osteosarcoma pts seen per year		
0–3	20	11%
4–7	67	37%
8–10	29	16%
10+	55	31%
Skipped	12	7%
Factor that Predominantly Guides Clinical Management Decision		
National Guidelines (e.g., NCCN)	78	43%
Institutional policies/standards	42	23%
Personal clinical practice and experience	51	28%
Skipped	12	7%

### Timing of resection

**Figure 1:**
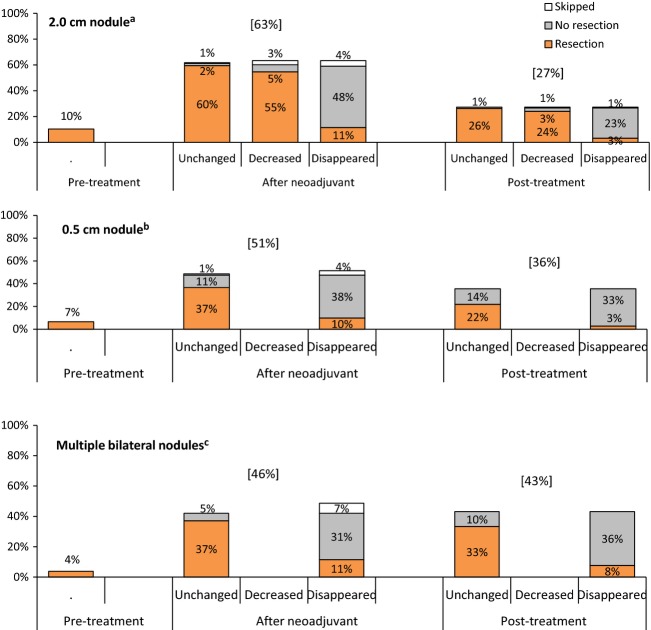
Timing of initiation of management and resection for 2.0 cm^a^, 0.5 cm^b^, and multiple bilateral^c^ nodules. ^a^*n = *183 survey responders. Significantly more 2.0 cm are managed at pretreatment than multiple bilateral (*P = *0.0235). ^b^*n* = 183 survey responders. Significantly less 0.5 cm are managed after neoadjuvant therapy than 2.0 cm (*P* = 0.03). ^c^*n* = 183 survey responders. Significantly less multiple bilateral nodules are managed after neoadjuvant therapy than 2.0 cm (*P* = 0.002). Decreased not option for the respondents in the case of 0.5 cm nodule or multiple bilateral nodules.

The majority of physicians (63%) chose to manage a unilateral 2 cm nodule after neoadjuvant chemotherapy was completed, whereas 10% chose to manage the nodule prior to initiation of treatment and 27% deferred management until completion of all planned chemotherapy. The percentage of respondents addressing the nodule at the time of completion of neoadjuvant chemotherapy decreased significantly when presented with the scenarios of 0.5 cm nodule and multiple bilateral nodules (51%, *P* = 0.03, and 46%, *P* = 0.002, respectively) (Fig.1).

**Figure 2:**
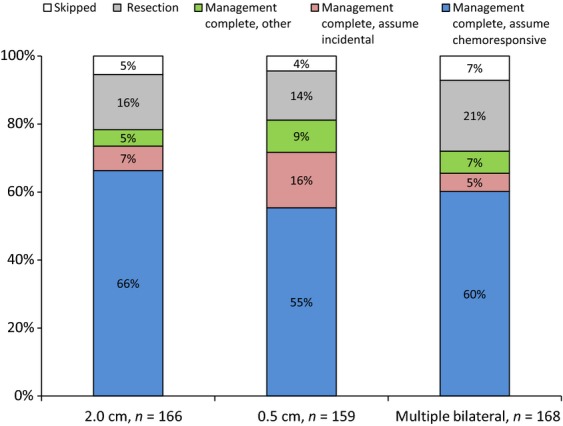
Reasons for not resecting after nodules disappeared after neoadjuvant therapy or posttreatment^a^.

If the survey participants chose to defer management of the nodules until after neoadjuvant chemotherapy or after completion of all planned chemotherapy, they were told that the primary tumor had >90% necrosis and presented with additional scenarios of the nodule remaining unchanged, decreased in size, or disappeared altogether. Of the 166 respondents who chose to defer management of a solitary 2 cm nodule, 78% believed management was complete when the nodule disappeared on repeat imaging. This practice was not statistically significantly different in the subcentimeter or multiple bilateral nodules scenarios (80% and 72%, respectively) (Figs.1 and 2).

The respondents who chose to forego exploration of the lung with the disappeared nodule were then asked their perception of nodule. The majority believed that the disappeared nodule represented chemoresponsive disease (66%, 55%, and 60%, in the 2 cm, 0.5 cm, and multiple bilateral nodules, respectively). In the 0.5 cm nodule scenario, there was a statistically significant increase in the percentage of respondents who believed that the disappeared nodule represented an incidental finding (16%, *P* = 0.015 and *P* = 0.002 when compared with the 2 cm (7%) and multiple bilateral nodules (5%) scenarios) (Fig.2).

### Surgical technique

Thoracotomy was the most preferred technique for resection across all scenarios. Thoracoscopy was the preferred modality (58%) only for the few respondents who chose to address the 2 cm and the 0.5 cm nodules in the pretreatment period. In the scenario of multiple bilateral nodules, most respondents, regardless of the scenario faced, expressed their preference for performing a thoracotomy. Thoracoscopy was never suggested when the nodules had completely disappeared (Table2).

**Table 2:** Surgical technique of resections performed; unchanged, decreased, and disappeared include responses from both after neoadjuvant and posttreatment

	Thoracoscopy	Thoracotomy	Sternotomy
2.0 cm			
Pretreatment, *n* = 19	58%	42%	0%
Unchanged, *n* = 120	21%	69%	10%
Decreased, *n* = 144	21%	73%	6%
Disappeared, *n* = 27	0%	78%	22%
0.5 cm			
Pretreatment, *n* = 12	58%	42%	0%
Unchanged, *n* = 107	25%^1^	69%	6%
Disappeared*, n* = 23	0%	78%	22%
Multiple bilateral			
Pretreatment, *n = 7*	29%	71%	0%
Unchanged, *n* = 129	12%^1^	77%	12%
Disappeared*, n* = 35	0%	80%	20%

1 Significantly more thoracoscopies performed in 0.5 cm nodules than multiple bilateral nodules (*P < *0.01).

**Figure 3:**
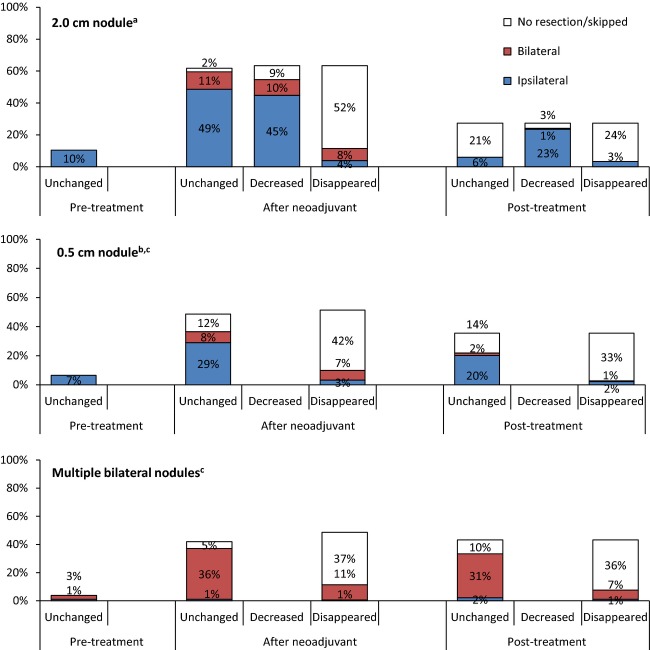
Contralateral lung exploration of total responders for 2.0 cm^a^, 0.5 cm^b^, and multiple bilateral^c^ nodules*. ^a^*n* = 183 survey responders. Significantly less 2.0 cm had contralateral exploration than multiple bilateral in after neoadjuvant unchanged (*P* < 0.0001), After neoadjuvant disappeared (*P* < 0.0001), posttreatment unchanged (*P* < 0.0001), and posttreatment disappeared (*P* = 0.0004). ^b^*n* = 183 survey responders. Significantly less 0.5 cm had contralateral exploration than multiple bilateral in after neoadjuvant unchanged (*P* < 0.0001), After neoadjuvant disappeared (*P* < 0.0001), posttreatment unchanged (*P* < 0.0001), and posttreatment disappeared (*P* = 0.0029). ^c^*n* = 183 survey responders. Decreased not option for the respondents in the case of 0.5 cm nodule or multiple bilateral nodules.

In every scenario in which the respondents resected the nodule, they were informed that the pathology was consistent with metastatic osteosarcoma. The participants were then questioned if they would consider surgery on the contralateral lung. Only in the scenario of multiple bilateral nodules did the majority of the respondents suggest performing exploration of the contralateral lung. This was a statistically significant difference when compared with both the 2 and 0.5 cm nodule scenarios regardless of the timing of the resection or the response to chemotherapy (Fig.3).

### Variation in management by specialty

When the management decisions were further analyzed by demographic data, clear differences were noted between medical and pediatric oncologists. Medical oncologists in every scenario were more likely to delay the timing of surgical resection until completion of all planned chemotherapy (38–53%) versus (10–23%) (Table3). Pediatric oncologists were significantly more likely than medical oncologists to explore the contralateral lung in both the 2 cm and the 0.5 cm scenarios, although a minority of respondents made that recommendation in these scenarios. Orthopedic surgeons did not significantly deviate in their management from pediatric or medical oncologists (data not shown). As noted before, the majority of respondents regardless of their specialty recommended contralateral lung resection in the scenario with multiple bilateral lung nodules (Table4).

**Table 3:** Initiation of management in posttreatment period between pediatric and medical oncologists

	Pediatric oncologist	Medical oncologist	Significance, *P-*value
2.0 cm^1^	10%	38%	0.004
0.5 cm^2^	23%	53%	0.005
Multiple Bilateral^3^	21%	45%	0.04

1 *n = *39 pediatric oncologists and 56 medical oncologists.

2 *n = *39 pediatric oncologists and 53 medical oncologists.

3 *n = *38 pediatric oncologists and 53 medical oncologists.

**Table 4:** Contralateral exploration of unchanged nodules between pediatric and medical oncologists; includes nodules managed both after neoadjuvant therapy and posttreatment; includes nodules managed both after neoadjuvant therapy and posttreatment

	Pediatric oncologist	Medical oncologist	*P-*value
2.0 cm^1^	19%	2%	0.01
0.5 cm^2^	28%	6%	0.04
Multiple bilateral^3^	100%	80%	0.12

1 *n = *32 pediatric oncologists and 51 medical oncologists who reported resections.

2 *n = *29 pediatric oncologists and 33 medical oncologists who reported resections.

3 *n = *32 Pediatric Oncologists and 40 medical oncologists who reported resections.

The number of years since completion of training revealed differences in the management of small nodules. Physicians who were practicing for more than 5 years versus physicians with less than 5 years experience were significantly more likely to recommend thoracotomy for the 2 cm nodules that had decreased in size after chemotherapy (77% vs. 48%, *P* = 0.0085) and for the 0.5 cm nodules (76% vs. 38%, *P* = 0.0057). There were no differences between these groups in the management of the 2 cm unchanged nodule and multiple bilateral nodules, with the majority of respondents preferring thoracotomy (Table5).

**Table 5:** Surgical technique of unchanged and decreased nodules between responders who completed training 0–5 years ago and 5+ years ago; includes nodules managed in both after neoadjuvant therapy and posttreatment

	0–5 years	5 years	*P-*value
2.0 cm			
Unchanged^1^			
Thoracoscopy	29%	22%	0.5743
Thoracotomy	67%	70%	0.8001
Decreased^2^			
Thoracoscopy	48%	17%	0.003
Thoracotomy	48%	77%	0.0085
0.5 cm			
Unchanged^3^			
Thoracoscopy	56%	19%	0.0032
Multiple bilateral			
Unchanged^4^			
Thoracotomy	38%	76%	0.0057
Thoracoscopy	18%	10%	0.398
Thoracotomy	76%	77%	1.000

1 *n* *=* 100 (0–5 years) and 80 (5+ years).

2 *n* *=* 21 (0–5 years) and 121 (5+ years).

3 *n* *=* 16 (0–5 years) and 90 (5+ years).

4 *n* *=* 17 (0–5 years) and 111 (5+ years).

All the other demographic data were examined, and no statistical significant differences were observed in the management of the scenarios presented (data not shown).

## Discussion

This survey presented three clinical scenarios to treating physicians with interest in malignancies of bone and demonstrated variability in the practice patterns in the management of pulmonary metastases in osteosarcoma. In all three scenarios, most respondents elected to manage the pulmonary nodules following neoadjuvant chemotherapy. A small minority of the respondents opted to resect the nodules prior to initiating neoadjuvant chemotherapy, and the remainder chose to resect at the end of planned chemotherapy.

Variability was demonstrated not only in the timing of resection, but also differences in the percentages were noted depending on the clinical scenario. This suggests that variability exists not only between the respondents, but also within the practice pattern of individual clinicians depending on the patient's presentation. Likewise, while thoracotomy was the preferred surgical modality for resection of the pulmonary nodules, the use of thoracoscopy varied based on the clinical scenario and the number of years in practice. Most respondents chose not to explore the contralateral lung unless radiographic evidence of bilateral disease was presented in the scenario with multiple bilateral nodules. In this practice there were differences between pediatric and medical oncologists, with a higher percentage of pediatric oncologists electing to explore the contralateral lung in the scenarios with the single lung nodule. In the situation that the nodule disappeared, most respondents elected to forgo exploration of the ipsilateral lung, attributing the change to chemoresponsive disease.

The data on the treatment of pulmonary metastases in osteosarcoma are limited to a few case series from which no definitive conclusions can be made. The most inclusive review of lung metastasectomy was presented by the International Registry of Lung Metastasis. This database included 5206 cases of varying histologies (42% sarcomas) collected from major centers of thoracic surgery and found that primary tumor type, disease-free interval (DFI), and the number of metastases are the most significant prognostic factors. Based on these variables, they developed prognostic grouping demonstrating that patients with resectable disease with a DFI greater than 36 months and a single metastasis had the best outcomes with those with unresectable disease having the worst 9. While this information establishes a baseline for risk-benefit assessment, it still contains the underlying limitations of a case series, further confounded by the difference in outcomes noted for the different histologies included. As this study did not randomize patients, it does not establish a survival advantage of surgery. The registry demonstrated variability in the accuracy of the preoperative imaging as well as in the surgical modalities utilized. Given the multiple variables, the authors could not make conclusions about the superiority of any surgical technique.

Without clear evidence to define management, practice patterns are based on national guidelines and expert opinion. The NCCN guidelines for metastatic osteosarcoma recommend surgery for all resectable disease. Likewise, the Children's Oncology Group protocols for patients with metastatic osteosarcoma recommend resection of all disease when deemed feasible. This survey is the first to assess practice patterns of treating physicians in the management of osteosarcoma lung metastases. In accordance with these guidelines, most of the clinicians surveyed advocated for resection of disease when it was still radiographically detectable. However, given the paucity of guidance, there was limited conformity in the practice patterns between the physicians and between the different clinical scenarios.

Similar variability in management of pulmonary metastasectomy was demonstrated by Internullo, et al. in a survey of members of the European Society of Thoracic Surgeons. The survey included metastasectomy of multiple tumor types, and found that the major areas of concern and contraindication for surgery were unresectable primary or unresectable metastatic disease. Consistent with the results from this survey, the majority of thoracic surgeons considered palpation mandatory, but had a more extensive list and increased variability of approaches utilized. The thoracic surgeons, like the respondents in this survey, demonstrated heterogeneity in their approach based on patient-specific variables, tailoring care based on presentation 10. The survey by Internullo et al. had a difference in scope and so did not inquire about timing of resection nor about differences in management based on the changes of the metastatic lesions during treatment. Internullo et al. also discuss that although a majority of the thoracic surgeons who responded recommended palpation, very few advocated for a bilateral approach.

Other factors not included in this survey may further increase the variability in the timing and method of resection of pulmonary metastases. The location of the primary tumor in this survey was uniform to minimize the effects of the definitive surgery as a confounding variable. However, a proximal humerus lesion may require further delay in metastasectomy to promote healing, whereas a chest wall primary, given the opportunity, may warrant metastasectomy at the time of definitive surgery, potentially leading to further variability in the management of the pulmonary nodules. One demographic factor that was not considered in this study was the geographic region of practice of the survey participants within the United States. Several regions within the country are associated with endemic fungal infections, and the prevalence of these pathogens may influence the incidence of pulmonary findings within the regional population. It would be of note if the geographic region of practice influenced management preferences of indeterminate lung nodules.

The major limitation of this study is the limitation of the instrument used. The respondents were presented with three contrived scenarios and limited choices to which to define their management preferences. Likewise, this survey relies on self-report, and as such may not accurately recapitulate real-world practice patterns. Many of the respondents provided very thoughtful comments reflecting the art of medicine beyond the scope of this questionnaire. They noted that the appearance, not just the size, of the nodules on the radiographic imaging may be more informative and affect their management choices. Many respondents commented that when the nodules disappeared on repeat imaging, they remained concerned and would recommend close surveillance with serial imaging. Lastly, the management of metastatic osteosarcoma requires a multidisciplinary effort, and the thoracic surgeons, who are a key component of that team, as well as the collaborative effort involved in the decision-making process were underrepresented in this survey.

In conclusion, this study found that there is variability in the reported management of pulmonary nodules in patients with osteosarcoma. Future studies need to address the uncertainty in the management of pulmonary metastases and prospectively collect data and evaluate if differences in timing, surgical technique, and exploration of the contralateral lung have implications for the outcomes of patients with osteosarcoma and pulmonary metastases.
